# Stabilization of the β-hairpin in Mason-Pfizer monkey virus capsid protein– a critical step for infectivity

**DOI:** 10.1186/s12977-014-0094-8

**Published:** 2014-10-30

**Authors:** Martin Obr, Romana Hadravová, Michal Doležal, Ivana Křížová, Veronika Papoušková, Lukáš Žídek, Richard Hrabal, Tomáš Ruml, Michaela Rumlová

**Affiliations:** Institute of Organic Chemistry and Biochemistry, Academy of Sciences of the Czech Republic, v.v.i., IOCB & Gilead Research Center, Flemingovo nám. 2, 166 10 Prague, Czech Republic; Department of Biochemistry and Microbiology, Institute of Chemical Technology, Technická 5, 166 28 Prague, Czech Republic; Laboratory of NMR Spectroscopy, Institute of Chemical Technology, Technická 5, 166 28 Prague, Czech Republic; CEITEC - Central European Institute of Technology, Masaryk University, Kamenice 5, 625 00 Brno, Czech Republic; Department of Biotechnology, Institute of Chemical Technology, Technická 5, 166 28 Prague, Czech Republic

**Keywords:** Retrovirus, Assembly, M-PMV, Capsid protein, Maturation, β-hairpin

## Abstract

**Background:**

Formation of a mature core is a crucial event for infectivity of retroviruses such as Mason-Pfizer monkey virus (M-PMV). The process is triggered by proteolytic cleavage of the polyprotein precursor Gag, which releases matrix, capsid (CA), and nucleocapsid proteins. Once released, CA assembles to form a mature core – a hexameric lattice protein shell that protects retroviral genomic RNA. Subtle conformational changes within CA induce the transition from the immature lattice to the mature lattice. Upon release from the precursor, the initially unstructured N-terminus of CA is refolded to form a β-hairpin stabilized by a salt bridge between the N-terminal proline and conserved aspartate. Although the crucial role of the β-hairpin in the mature core assembly has been confirmed, its precise structural function remains poorly understood.

**Results:**

Based on a previous NMR analysis of the N-terminal part of M-PMV CA, which suggested the role of additional interactions besides the proline-aspartate salt bridge in stabilization of the β-hairpin, we introduced a series of mutations into the CA sequence. The effect of the mutations on virus assembly and infectivity was analyzed. In addition, the structural consequences of selected mutations were determined by NMR spectroscopy. We identified a network of interactions critical for proper formation of the M-PMV core. This network involves residue R14, located in the N-terminal β-hairpin; residue W52 in the loop connecting helices 2 and 3; and residues Q113, Q115, and Y116 in helix 5.

**Conclusion:**

Combining functional and structural analyses, we identified a network of supportive interactions that stabilize the β-hairpin in mature M-PMV CA.

**Electronic supplementary material:**

The online version of this article (doi:10.1186/s12977-014-0094-8) contains supplementary material, which is available to authorized users.

## Background

Formation of a mature, fully infectious retroviral particle encompasses two distinct steps: (i) assembly of an immature, roughly spherical particle from Gag-derived polyprotein precursors and (ii) proteolytic cleavage of the polyprotein precursors, leading to reorganization of the immature particle interior and formation of a mature core.

The immature particle of a retrovirus such as Mason-Pfizer monkey virus (M-PMV) consists of approximately 1500–2500 Gag molecules that are self-associated into a hexameric lattice. Maturation of the immature particle takes place during or shortly after budding through the host cell membrane. The viral protease is autocatalytically activated and cleaves Gag into the individual structural proteins matrix (MA), capsid (CA), and nucleocapsid (NC). Proteolytic cleavage initiates changes in the CA structure, enabling subsequent formation of a mature hexameric lattice that forms the viral core. Because the mature CA shell protects the viral genomic RNA, proper rearrangement of CA allowing correct assembly of CA proteins is crucial for the viral life cycle.

Retroviral capsid proteins consist of two helical domains, the N-terminal domain (NTD) and C-terminal domain (CTD), connected by a short flexible segment. Despite low sequence homology among CAs from different retroviruses, the structural arrangement of both CA domains is highly conserved [1-7]. The NTD consists of an N-terminal β-hairpin followed by six or seven α-helices, and the CTD consists of four α-helices. Interactions between CA domains, i.e., NTD-NTD, CTD-CTD, and NTD-CTD, are crucial for assembly. Interestingly, the structural roles of the two domains are exchanged in the immature and mature hexameric lattices. While immature intra-hexameric contacts are formed predominantly by CTD-CTD interactions, the mature hexameric unit is held together by NTD-NTD interactions [8,9].

The NMR structure of the N-terminal 283-amino-acid fragment of HIV-1 Gag, comprising MA and CA-NTD, confirmed the hypothesis that the region corresponding to the β-hairpin is unfolded in the Gag polyprotein and that β-hairpin formation is triggered upon complete proteolytic cleavage and release of CA from Gag [10]. Comparing the structures of mature HIV-1 CA-NTD and HIV-1 CA-NTD N-terminally extended to MA revealed that β-hairpin formation induces negligible structural changes, including a small displacement of helix 6 (residues 112–117 in HIV-1 CA) [10,11]. One of the initially suggested roles of the β-hairpin was formation of a β-barrel-like structure stabilizing mature hexamers. However, the electron cryo-crystallography map of HIV-1 CA showed that the individual strands are too far separated to mediate direct interactions stabilizing the mature CA hexamer [12]. Currently, it is assumed that the β-hairpin plays a role in destabilization of the immature lattice and formation of CA interfaces, mediating the crucial interactions for stabilization of the mature lattice [10-14].

The length of the N-terminal β-hairpins varies slightly in different retroviruses. However, this structural motif is invariably stabilized by a salt bridge between the N-terminal proline and an aspartate or glutamate residue in helix 3 [1,2,5,6,15]. In M-PMV, the β-hairpin is stabilized by a salt bridge between the N-terminal proline (P1) and D57 [6]. Mutating these two residues in a proviral M-PMV DNA resulted in altered M-PMV Gag processing, and the mutated viral particles released from the transfected cells had aberrant cores and were non-infectious [16]. Similarly, any modifications of M-PMV CANC, a Gag-derived protein that mimics the assembly of mature particles, affecting β-hairpin formation (e.g., deletion [ΔProCANC], mutation of P1 or D57, or N-terminal extension of CA by one or more amino acids) resulted in the inability of CANC to form mature-like tubular structures in an *in vitro* assembly assay. Instead, immature-like, spherical virus-like particles (VLPs) were formed [16,17]. The situation is different in the case of HIV-1 ΔProCANC. Even though deletion of the N-terminal proline should prevent β-hairpin formation, HIV-1 ΔProCANC is still able to assemble into tubular, mature-like particles [18]. In contrast, deletion of the N-terminal proline in the Rous sarcoma virus (RSV) CANC protein (ΔProCANC) resulted in an inability to assemble into any regular structure [19]. Both HIV-1 and RSV require N-terminal extensions to CA to initiate assembly of immature-like particles [19-21].

Based on the NMR structure of M-PMV CA-NTD, we previously identified residues other than P1 and D57 that are potentially important in stabilization of the β-hairpin relative to the α-helical core [6]. These residues comprise R14 and H16 in the β-hairpin and D111 and D117 in helix 5.

In our present work, we studied the necessity of additional β-hairpin stabilization for mature particle formation. Mutations in the β-hairpin and helix 5 of CA-NTD were introduced into the M-PMV sequence, and their impact on the assembly of mature particles was investigated. Both *in vitro* and *in vivo* experiments confirmed the critical role of residue R14 in the β-hairpin, W52 located in the loop connecting helices 2 and 3, and residues Q113, Q115, and Y116 in helix 5. Comparing the chemical shifts in NMR spectra of R14K and WT CA-NTD showed that the mutation caused changes only in the vicinity of helix 5. Mutation of R14 as well as mutation of residues in helix 5 resulted in aberrant cleavage of CA during M-PMV maturation. Comparison of hydrogen-deuterium exchange of R14K and WT CA-NTD showed that the R14K mutation leads to an increase in β-hairpin flexibility, resulting in exposure of a novel CA cleavage site.

## Results

### In vitro assembly

The NMR structure of M-PMV CA-NTD is composed of six α-helices and an N-terminal β-hairpin [6]. The N-terminal β-hairpin is stabilized by a salt bridge between P1 and D57 in helix 3. Besides the salt bridge, most residues involved in the stabilization of the β-hairpin lie in helix 5 [6].

**Figure 1:**
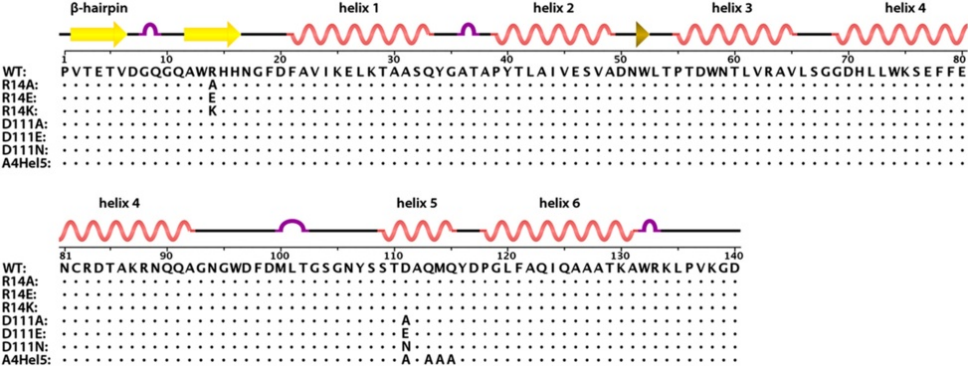
**Schematic representation of M-PMV CA-NTD.** Amino acid sequences of M-PMV CA-NTD wild type (WT) and mutants (R14A, R14E, R14K, D111A, D111E, D111N, A4Hel5) used in this study. The positions of secondary structure elements are annotated above the sequence.

**Figure 2:**
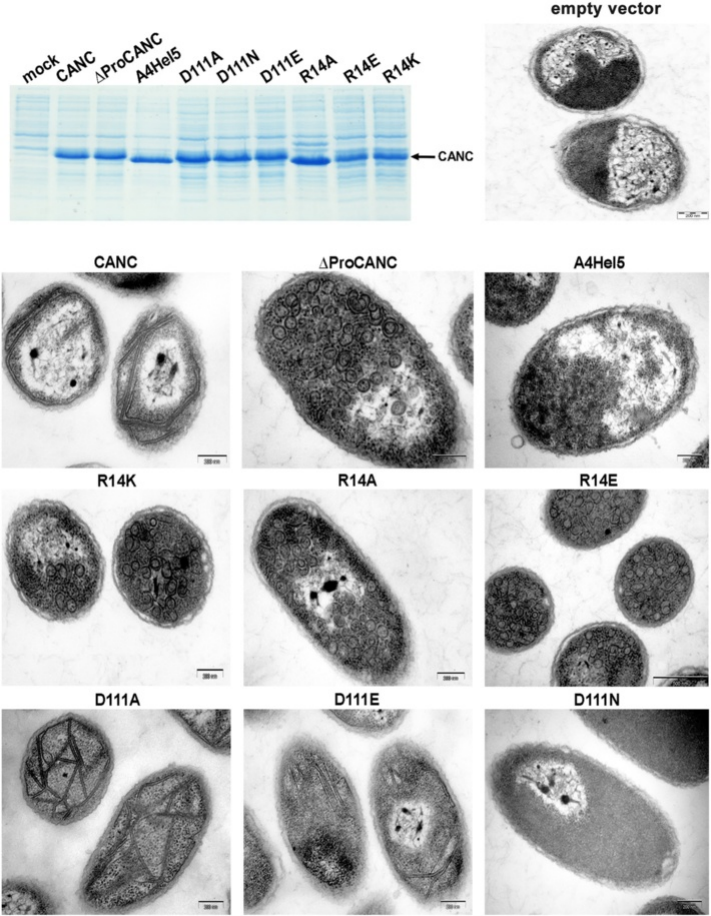
**Expression and morphology of mutant M-PMV CANC in** ***E.coli*** **.** Coomassie blue stained SDS-PAGE gel of *E. coli* expressing WT and mutant M-PMV CANC proteins (upper left panel). Electron micrographs of thin sections of *E. coli* expressing WT and mutant M-PMV CANC. Bars: 200 or 500 nm.

To analyze the importance of helix 5 in stabilization of the β-hairpin, we prepared a mutant, termed A4Hel5, in which four helix 5 residues (D111, Q113, M114, Q115) were replaced with alanines (Figure 1). Due to the spatial proximity of residue R14 in the β-hairpin and D111 in helix 5, an electrostatic interaction between these two residues was proposed to additionally stabilize the β-hairpin [6]. To analyze the importance of this putative interaction, we prepared a series of mutations, exchanging both residues for small neutral, isopolar and oppositely charged residues (i.e., R14A, R14K, R14E and D111A, D111E, D111N) (Figure 1). All mutations were introduced into a bacterial expression vector carrying M-PMV CANC (*CANCpET22b*), the proteins were expressed in *E. coli* BL21 (DE3) (Figure 2), and transmission electron microscopy (TEM) analysis of bacterial thin-sections was carried out (Figure 2). The M-PMV CANC and ΔProCANC proteins have previously been shown to assemble into tubular, mature-like particles or roughly spherical, immature-like particles, respectively [17,18,22]. TEM analysis of bacterial cells expressing the mutant CANC proteins revealed three different phenotypes: D111A and D111E formed WT-like tubular structures similar to those observed for mature-like CANC; D111N and A4Hel5 failed to assemble into any regular structure; and R14A, R14K, and R14E assembled into particles of spherical appearance similar to those observed for immature-like ΔProCANC particles. These data suggest that residues 110–115 in helix 5 play an important role in assembly.

### M-PMV particle production, release, processing, morphology, and infectivity

**Figure 3:**
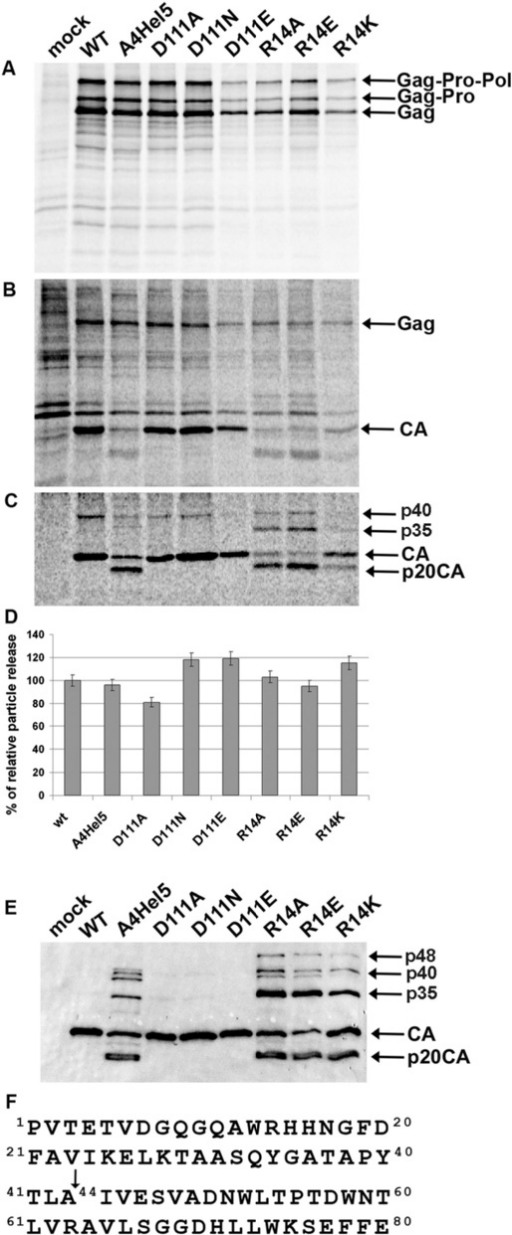
**Synthesis, release, and processing of WT and mutant M-PMV CA-NTD.** HEK 293 T cells were transfected with WT or mutant M-PMV CA-NTD proviral DNA. Viral proteins were metabolically labeled with a [^35^S] cysteine-methionine mix for 30 min and chased for 16 h. M-PMV CA-related proteins were then immunoprecipitated from the cells and culture media and analyzed by SDS-PAGE: **(A)** Intracellular M-PMV proteins Gag (Pr78), Gag-Pro (Pr95), and Gag-Pro-Pol (Pr180) immunoprecipitated from cell lysate after 30 min pulse, and **(B)** after 16 h chase. **(C)** CA-derived proteins of released M-PMV particles were immunoprecipitated from the culture media 16 h after the chase. **(D)** Quantification of WT and mutant M-PMV particle release from HEK 293 T cells. Band intensities of [^35^S] pulse–labeled Gag (Pr78) and released CA were calculated. The relative percent of CA released into the culture media was corrected for intracellular expression of individual samples. **(E)** Western blot analysis of released WT and mutant M-PMV CA-NTD particles. VLPs from the culture media were collected by centrifugation through a 20% sucrose cushion 48 h post-transfection. The viral proteins were analyzed by SDS-PAGE, blotted onto a nitrocellulose membrane, and detected with rabbit antibodies raised against M-PMV CA. **(F)** Amino acid sequence of 80 N-terminal amino acids of M-PMV CA. The arrow indicates the internal cleavage between A^43^ and I^44^ identified by N-terminal amino acid sequencing.

Next, we studied the impact of the mutations on the M-PMV life cycle. The mutations were introduced into a proviral M-PMV vector (*pSARM4*). To analyze the effect of the mutations on expression and release of viral particles, HEK 293 T cells were transiently transfected with the WT and mutant proviral vectors, and expression of polyprotein precursors and virus release were evaluated in pulse-chase experiments. At 48 h post-transfection, the proteins were metabolically labeled with ^35^S and chased for 4 h. Viral particles released into the cultivation media were collected by centrifugation through a sucrose cushion. Both intracellular proteins and those released into the medium were immunoprecipitated with an anti-M-PMV CA antibody. Gag, Gag-Pro, and Gag-Pro-Pol polyprotein precursors were detected in all samples (Figure 3A). Mature CA protein was present in viral particles released from cells expressing the WT protein and the D111 mutants (D111A, D111N, and D111E; Figure 3B, C). In addition to properly processed CA, an internal cleavage product with a molecular weight of approximately 20 kDa (p20CA) was detected in virions released from cells expressing A4Hel5 or R14 mutants (R14A, R14K, and R14E; Figure 3C). No significant block of virus release, calculated from relative cell- and virion-associated protein levels, was observed for any of the mutants (Figure 3D). To confirm impaired maturation, Western blot analysis of pelleted viral particles released from cells transfected with the mutants was performed (Figure 3E). Indeed, in contrast to the WT and D111 mutants, in which we detected only properly processed CA, we observed inefficient Gag processing in A4Hel5 and R14 mutants, resulting in CA containing proteins of higher molecular weight (Figure 3E). Moreover, the internal cleavage product p20CA was observed in the A4Hel5 and R14 mutants (Figure 3E). By performing Edman degradation of p20CA, we identified this newly created cleavage site as ^39^PYTLA*IVESV^48^, located within helix 2 of the CA protein (Figure 3F). The 20-kDa product resulting from internal CA cleavage thus corresponds to the fragment ^44^IVESV-GLAM^226^.

**Figure 4:**
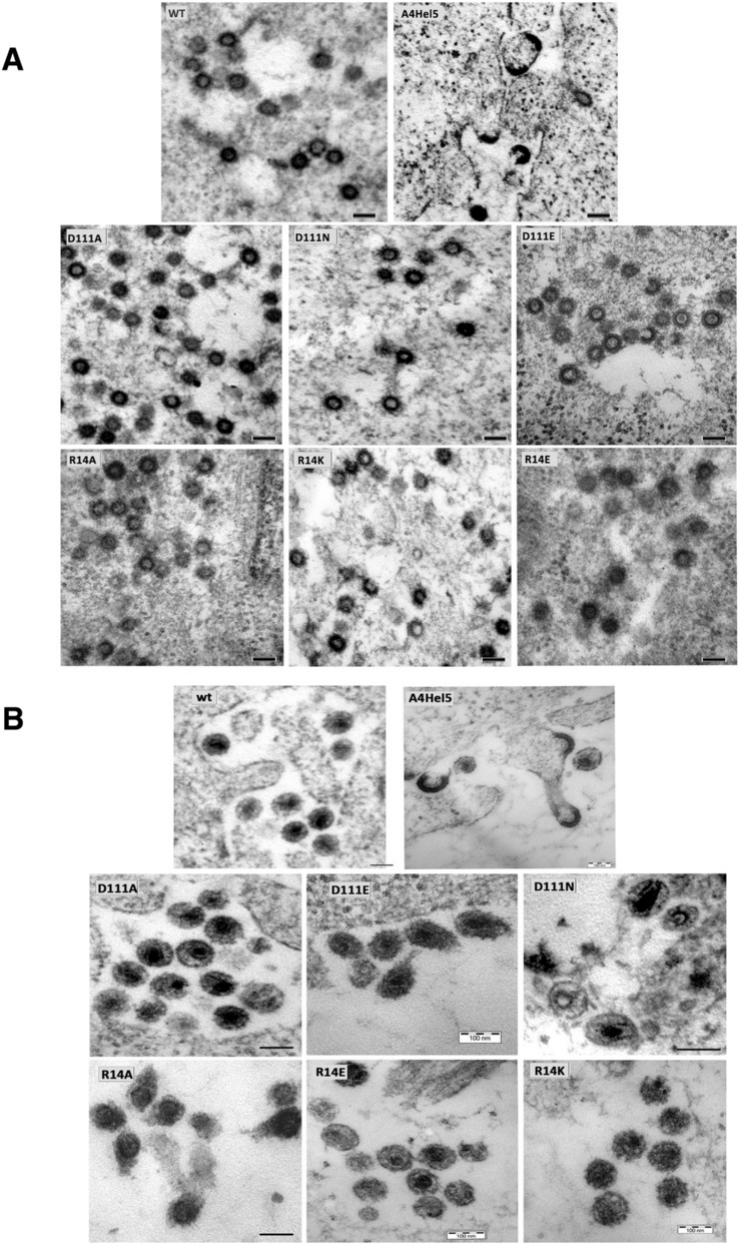
**Transmission electron microscopy images of HEK 293 T cells expressing WT and mutant M-PMV CA-NTD.** At 48 h post-transfection of HEK 293 T cells with WT or mutant proviral vector, the cells were fixed in glutaraldehyde and postfixed in 1% osmium tetroxide. The sections were contrasted with uranyl acetate and lead citrate and analyzed using a JEOL JEM-1200EX analytical transmission electron microscope: **(A)** Immature particles assembled within the cells. **(B)** Mature viruses released from the cells. Bars: 100 nm.

To analyze the effect of D111, R14, and A4Hel5 mutations on formation of immature and mature M-PMV particles, HEK 293 T cells were transiently transfected with WT and mutant proviral vectors. The cells were fixed, and ultrathin sections of the cells were analyzed using TEM (Figure 4). Similar to WT, all the mutants assembled their immature particles within the cytoplasm of the cells. The only exception was the A4Hel5 mutant, which did not assemble typical intracytoplasmic particles. Instead, only C-type-like particles accumulated underneath the plasma membrane (Figure 4A). In the released particles, cores resembling those formed by WT were observed for the D111 mutants. In contrast, aberrant core-like structures were found in released A4Hel5 and R14 mutant virions (Figure 4B).

**Figure 5:**
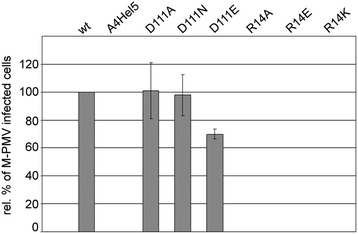
**Relative infectivity of M-PMV CA-NTD mutants determined by single-round assay.** HEK 293 T cells were co-transfected with WT or mutant *pSARM-EGFP* and *pTMO* vectors. At 48 h post-transfection, viruses were filtered from culture media and normalized by quantitative Western blotting for CA. Equivalent amounts of viruses were used to infect fresh HEK 293 T cells. At 48 h post-infection the cells were harvested and the numbers of GFP-positive cells were determined by flow cytometry (BD FACSaria). The mean percentage of five independent infectivity measurements (with calculated standard deviations) for each mutant relative to the WT is shown.

Next, we analyzed the effect of the D111, R14, and A4Hel5 mutations on M-PMV infectivity in a single-round infectivity assay [23]. The mutations were introduced into the *pSARM-EGFP* vector, which carries the entire M-PMV genome except for the *env* gene, which is replaced with *EGFP*. This plasmid was co-transfected with an Env-expression vector (*pTMO*) [24] into HEK 293 T cells. Virions released into cell culture supernatants were normalized for CA content and used for infection of fresh HEK 293 T cells. At 48 h post-infection, the cells were analyzed by flow cytometry (Figure 5). The relative infectivity of the WT was considered 100%. Except for the D111E mutant, whose infectivity decreased by approximately 30% compared to WT, the other D111 mutations had no effect on M-PMV infectivity. In contrast, A4Hel4 and all three R14 mutants were completely noninfectious.

### Structural analysis of D111N and R14K CA-NTD

**Figure 6:**
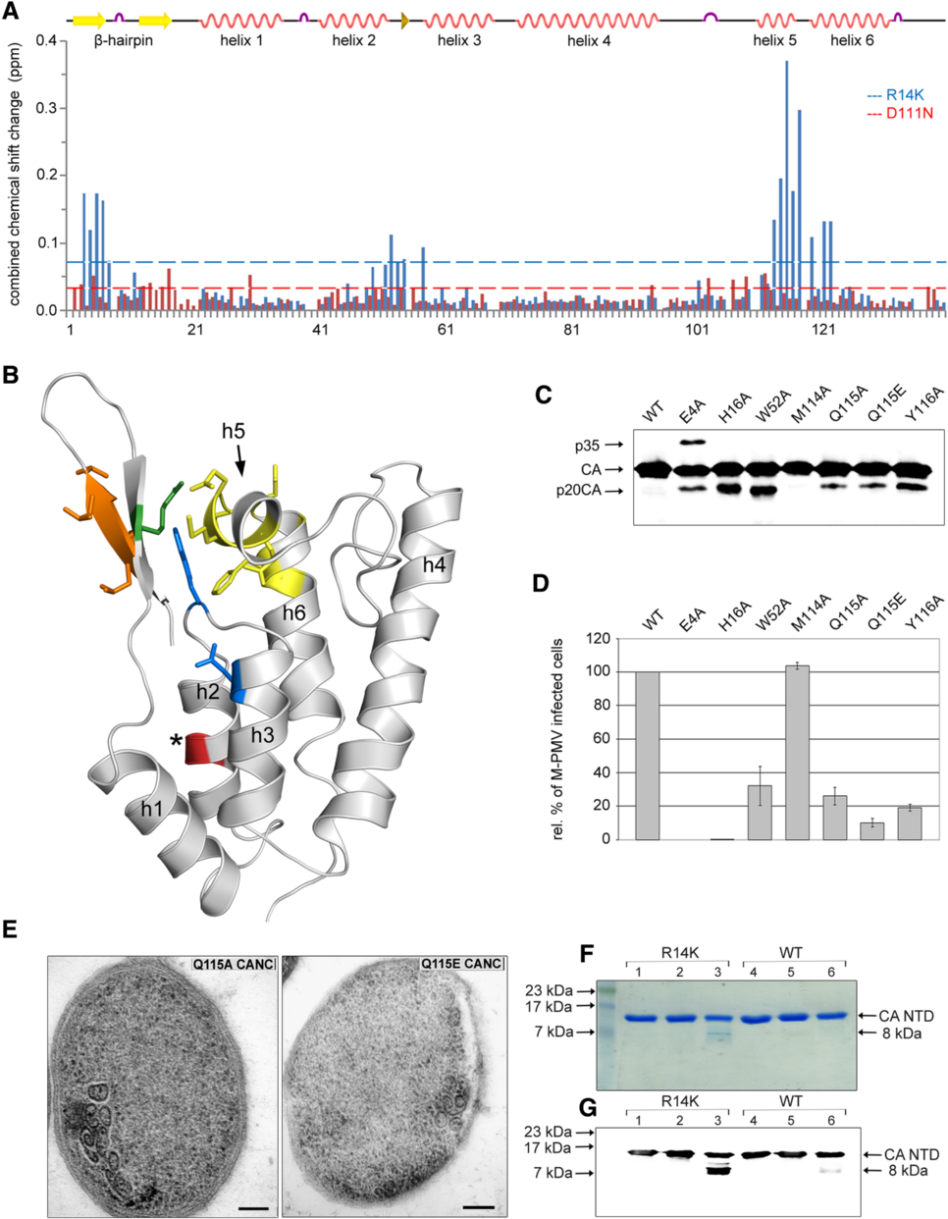
**Impact of R14K and D111N mutations on the CA-NTD structure. (A)** CCS Combined chemical shift (CCS) changes of R14K and WT CA-NTD (blue), and D111N and WT (red). Helices 1 - 6 are annotated as h1 - h6. **(B)** Residues with the largest CCS caused by the R14K mutation: green - R14; orange: T3, E4, T5, V6 in β-hairpin; blue: W52, D57 in loop connecting helices 2 and 3; yellow: Q113, M114, Q115, Y116, D117, G119, F121, A122 in helices 5, 6, and the connecting loop. The asterisk indicates the internal cleavage site between A43 and I44 (red). The WT M-PMV CA-NTD structure (PDB ID: 2KGF) was used for this model. **(C)** Western blot of CA related proteins in released WT and CA-NTD mutant M-PMV particles detected with rabbit antibodies raised against M-PMV CA. VLPs released from the of HEK 293T cells 48 h post-transfection were centrifuged through a 20% sucrose cushion. **(D)** HEK 293T cells were co-transfected with WT or mutant pSARM-EGFP and pTMO vectors. At 48 h post-transfection, the virus was filtered from culture media and normalized by quantitative Western blotting for CA. At 48 h post-infection with equivalent amounts of virions, the HEK 293T cells were harvested and the GFP-positive cells were counted using flow cytometry. The mean percentage of three independent infectivity measurements for each mutant relative to WT is shown. **(E)** Electron micrographs of thin sections of E. coli expressing Q115A and Q115E M-PMV CANC protein. **(F, G)** Coomassie blue stained **(F)** or western blot analysis **(G)** of R14K CA-NTD and WT CA-NTD proteins cleavage in vitro. M-PMV R14K CA-NTD and WT CA-NTD purified proteins in storage buffer (lanes 1 and 4, respectively) were diluted into M-PMV protease cleavage buffer (lanes 2 and 5). M-PMV protease was added to R14K CA-NTD (lane 3) and WT CA-NTD (lane 6) and incubated for 4 h at 37°C.

To assess the importance of residues R14 and D111 in stabilization of the β-hairpin, we performed NMR structural analysis of D111N and R14K CA-NTD mutants. The respective mutations were introduced into a CA-NTD expression vector (*CA-NTDpET22b*), and the corresponding ^13^C and ^15^N double-labeled proteins were expressed in *E. coli* BL21 (DE3) cells. The proteins were purified by gel chromatography and concentrated to 2 mM. The ^1^H-^15^N HSQC spectra of both proteins were well-dispersed, with the majority of cross-peaks superimposable on the WT spectrum, confirming that both proteins were properly folded and that their overall folds did not differ from that of WT. To assign chemical shifts of the backbone amide group atoms, three triple-resonance experiments (HNCA, HNCACB, CBCA(CO)NH) and HCCH-TOCSY were conducted (Additional file 1: Figure S1). For the D111N mutant, 125 backbone amide resonances of 135 possible were assigned, and for the R14K mutant, 119 resonances were assigned. Residues that were not assigned in the WT CA-NTD (i.e., G8, Y40, G68, G69, N106, and T110) [6] could not be assigned in the spectra of the mutant proteins either. In addition to those, the following residues were not assigned in the D111N mutant: T37, G93, G95, and N111; and in the R14K mutant: V2, W13, K14, H15, H16, N17, G18, F19, D20, and F21. The increased number of missing resonances in the N-terminus of R14K mutant is probably caused by β-hairpin impairment and slow conformational exchange. This strengthens our model of increased β-hairpin flexibility in the R14K mutant. Because the chemical shifts of the backbone amides of D111N CA-NTD were very similar to those of WT (Figure 6A, red), we measured the ^15^N-edited NOESY spectrum of the mutant and compared it with the WT spectrum. Long range NOE contacts of close neighbors of N111 and residues from the β-hairpin showed no difference compared with the corresponding contacts in WT (data not shown). Based on these data, we concluded that the effect of the D111N mutation on the structure of CA-NTD is negligible.

Comparison of the available amide combined chemical shifts (CCS) of R14K CA-NTD to those of WT revealed that the mutation had an effect on the chemical environment of 14 residues (Figure 6A, blue). These residues are localized in or close to helix 5 (Q113, M114, Q115, Y116, D117, G119, F121, A122), in the β-hairpin (T3, E4, T5, V6), and in the proximity of helix 3 (W52, D57). Interestingly, the residues affected by the R14K mutation are closely clustered in the CA-NTD domain (Figure 6B). To analyze whether these residues participate in β-hairpin stabilization, several of them (i.e., E4 in the β-hairpin; W52 in the loop connecting helix 2 and 3; and M114, Q115, and Y116 in helix 5; and additionally, H16 residue selected on the basis of its proximity to the aforementioned residues in the NMR structure of the WT CA-NTD) were mutated to alanine. We determined the impact of these mutations on M-PMV CA processing and infectivity of the virus (Figure 6 C,D). An internal cleavage of CA, similar to that detected in the R14 and A4Hel5 mutants, was observed in the E4A, H16A, W52A, Q115A, Q115E, and Y116A mutants, but not in M114A (Figure 6C). Decreased infectivity was observed for all the mutant viruses except the M114A mutant (Figure 6D). To analyze the impact of the Q115A and Q115E mutations on assembly in *E. coli,* we expressed Q115A and Q115E CANC mutants in *E. coli* BL21 (DE3). TEM analysis showed that the Q115A and Q115E CANC mutants (Figure 6E), similar to the R14 mutant (Figure 2), failed to assemble the mature-like tubular structure. Only immature-like spherical or aberrant particles were observed (Figure 6E). To test our model supposing that increased conformation flexibility of the β-hairpin results in alternative cleavage, we compared the pattern of products generated by the in vitro cleavage of R14K CA-NTD mutant protein with that of WT CA-NTD protein (Figure 6F, G). Samples of purified R14K and WT CA-NTD proteins were incubated with M-PMV protease and cleavage products were separated by SDS-PAGE gel and either stained by Coomassie blue (6 F) or transblotted and developed immunochemically using anti-M-PMV CA antibody (Figure 6G). A cleavage product of R14K CA-NTD proteins with an estimated molecular weight 8 kDa was observed after the 4 h incubation of the sample with M-PMV protease. Protein sequencing confirmed the N-terminus of the 8 kDa cleavage product (IVESV) to be identical to that of the p20 protein in the virus particles carrying R14 and A4Hel5 mutations. In contrast to the R14K CA-NTD protein, WT CA-NTD remained uncleaved and only subtle 8 kDa cleavage product was visible on western blot.

**Figure 7:**
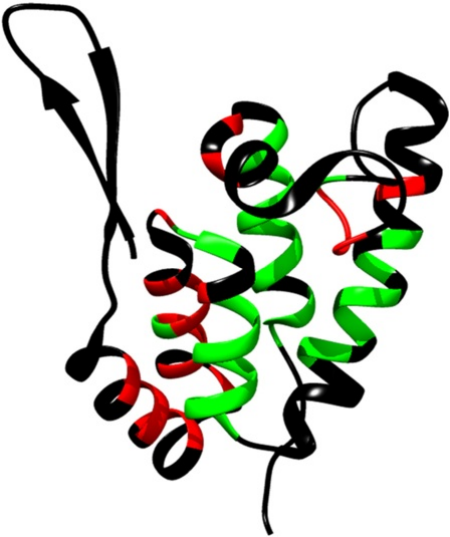
**Comparison of hydrogen-deuterium exchange rates for WT and R14K CA-NTD proteins.** The amide proton relative exchange rates of individual segments of the protein backbone are shown in different colors: black - solvent-exposed amide groups exchanged protons prior to measurement, green - comparable relative rate of exchange for both WT and R14K proteins, red - substantially higher rates of exchange for the R14K mutant than for the WT protein.

These results strengthened our hypothesis that impairment of the β-hairpin–helix 5 stabilizing interactions leads to an increase in β-hairpin flexibility, resulting in relocation of helix 1, which causes exposure of a novel CA cleavage site. Therefore, we analyzed the backbone solvent accessibility by measuring the hydrogen-deuterium exchange rate for R14K and WT CA-NTD (Figure 7). The CA-NTD residues were grouped according to the relative exchange rates of their amide protons. The first group exchanged amide protons prior to measurement (Figure 7, black color), the second group exchanged protons with a relative rate comparable for both WT and R14K (Figure 7, green color), and the third group exchanged protons at a relatively higher rate for R14K than WT (Figure 7, red color). Residues with altered exchange rates were all situated in the proximity of helices 1, 2, and 5, thus supporting the hypothesis that these areas have different degrees of flexibility in WT and R14K.

## Discussion

The transition from an immature to mature lattice is controlled by changes in the CA structure. Based on hydrogen-deuterium exchange experiments, the structural changes that occur during HIV-1 CA maturation after proteolytic release of its N- and C-termini are formation of the β-hairpin, slight reorientation of helix 1, dissolution of a 3-fold interaction involving the region around the cyclophilin A-binding loop, and formation of the NTD-CTD interface [14]. Release of the N-terminus of HIV-1 CA leads to formation of the β-hairpin, a structural element critical for formation of a mature core [15]. Release of the C-terminus of HIV-1 or RSV CA proteins and subsequent structural changes are the final steps of Gag maturation, and they seem to be important for transition of the immature-like lattice into the mature virus particle [25]. Interestingly, it was shown that the β-hairpin is only created upon proteolytic cleavage of the HIV-1 CA C-terminus at the CA-SP1 site [14]. Based on the available mutational data, it is clear that prevention of β-hairpin formation or its stabilization *via* the proline-aspartate salt bridge are fatal for mature lattice formation of the infectious core of HIV-1 and M-PMV [15,16,26,27]. Although several functions of the β-hairpin have been proposed, its exact role remains unclear. One potential role of the β-hairpin is to contribute to destabilization of the immature lattice [10-14]. This assumption is supported by the fact that there is no evidence for direct intermolecular contacts mediated by the β-hairpin in the HIV-1 CA mature hexamer [9]. For equine infectious anemia virus (EIAV) CA, it was proposed that the purpose of maturational folding of the β-hairpin is to extend helix 1 at the N-terminus to enhance CA oligomerization [1]. Our present work supports another hypothesis – that β-hairpin formation and stabilization serve as a mechanism restricting CA-NTD flexibility, thus locking the CA-NTD helical bundle in its mature conformation [14].

Since the structure of the mature M-PMV core is not available, we used the NMR structure of M-PMV CA-NTD to demonstrate that the β-hairpin is additionally stabilized by interactions with residues localized in helix 5 (residues 110–115) [6]. We designed a series of mutations to abolish these putative stabilizing interactions. Initially, we exchanged four amino acids in helix 5 (^111^**D**A**QMQ**^115^) for alanines (A4Hel5). Subsequently, residues R14 and D111, which were proposed to form stabilizing electrostatic interactions, were exchanged for similar (R14K, D111E), neutral (R14A, D111A), or oppositely charged (R14E, D111N) residues. *In vitro* and *in vivo* analyses of these mutants revealed a critical role of residue R14 in the formation of mature particles. Even the exchange of arginine to lysine led to a complete loss of infectivity and inability of the R14K CANC mutant to assemble into mature-like structures *in vitro*. This is likely due to the inability of R14 mutants to lock CA-NTD in β-hairpin-stabilized mature conformation, similarly to ΔProCANC particles, in which β-hairpin cannot be formed. Notably, all mutations of R14 tested, as well as the A4Hel5 mutation, induced formation of a novel internal cleavage site within helix 2. On the other hand, the D111 mutations did not impair the mature core assembly and did not dramatically change the infectivity. The only exception was the D111N mutant, which failed to assemble VLPs in *E. coli* cells. Since this mutation did not influence either infectivity or proteolytic processing in tissue culture cells, we consider this phenotype to be a “false negative” that can be ascribed to the somewhat artificial *in vitro* assembly system.

Our results show that residue R14 and residues 112–115 in helix 5 are indispensable for formation of mature particles. In contrast, the importance of the electrostatic interaction of residues R14 and D111 in the stabilization of the β-hairpin was not confirmed. The impacts of the D111N and R14K mutations on the structure of CA-NTD were analyzed using NMR spectroscopy. Chemical shifts in ^1^H-^15^N HSQC spectra of both CA-NTD mutants showed that the overall protein fold was not affected by either mutation. Moreover, chemical shift differences between the D111N mutant and WT and comparison of ^15^N-NOESY spectra showed that the structural changes caused by the mutation are negligible. In contrast, the ^1^H-^15^N HSQC spectrum of the R14K CA-NTD mutant showed more profound changes in chemical shifts compared to the WT. The most dramatic changes were found in both the β-hairpin and helix 5. Mutational analyses showed that the phenotypes of viruses in which the residues most affected by R14 mutation were mutated (i.e., E4A, H16A, W52A, Q115A, Q115E, and Y116A) were similar to that of the R14K mutant, i.e., CA-NTD was internally cleaved and the viruses were less infectious. Similar to R14K CANC, the Q115A and Q115E CANC mutant proteins also failed to assemble into tubular-like mature structures in *E. coli.* We hypothesize that these mutations disrupted the network of interactions stabilizing the β-hairpin. This subsequently increased the flexibility of helix 1, thus exposing a new cleavage site for M-PMV protease (Figure 6B).

Hydrogen-deuterium (H/D) exchange monitored by mass spectroscopy experiments showed that the transition from the immature to mature lattice of HIV-1 Gag is accompanied by increased protection against H/D exchange due to decreased flexibility in helix 1 and the β-hairpin [14]. This data is in good agreement with our H/D exchange results, which show the same shift in H/D protection pattern when comparing R14K, a CA-NTD mutant destabilizing the β-hairpin and helix 5 interactions, with WT CA-NTD. Taken together, our observations support a model in which the main importance of the β-hairpin for mature assembly lies in maintaining tight packing of helices 1–3, facilitating central 18-helix bundle formation in mature particles. We show that in M-PMV CA this packing effect is mediated not only by β-hairpin formation itself, but also by its interaction with helix 5.

## Conclusions

Upon maturation, the N-terminus of retroviral CA folds to form a β-hairpin, which is primarily stabilized by a salt bridge between the N-terminal proline and a highly conserved aspartate residue. Combining functional and structural analyses, we identified a network of supportive interactions stabilizing the N-terminal β-hairpin in mature M-PMV CA-NTD. This network comprises residues in the β-hairpin, the loop connecting helices 2 and 3, and helix 5. Mutations of these residues most probably lead to an increase in β-hairpin flexibility and exposure of a novel CA cleavage site. Subsequently, despite the presence of certain level of the full-length CA, the concurrent occurrence of N-terminally truncated CA (p20) protein interferes with the formation of a mature core and this dominant-negative effect results in formation of aberrant core hampering the infectivity of the mutant virus. In summary, we show that in addition to the salt bridge between the N-terminal proline and aspartate 57, other β-hairpin-stabilizing interactions in M-PMV CA-NTD are essential for the virus infectivity.

## Methods

### Cloning

All DNA manipulations were carried out using standard subcloning techniques, and plasmids were propagated in *E. coli* DH5α. All newly created constructs were verified by DNA sequencing. To introduce point mutations within CA of a proviral M-PMV DNA vector (*pSARM4*), we initially used a helper vector prepared by ligation of a *Sac*I-*Eco*72I fragment corresponding to nucleotides 1165 to 3275 of M-PMV into *pUC19* (*MHelppUC19*) [23]. CA point mutations were created by PCR mutagenesis using primers carrying appropriate mutations (A4Hel5, R14A, R14K, R14E, D111A, D111N, D111E, E4A, H16A, W52A, M114A, Q115A, Q115E, Y116A) and suitable restriction endonuclease sites (*Stu*I for R14A/K/E; *Xma*I for D111A/N/E and A4Hel5; *Sal*I for E4A; *Kas*I for H16A; *Mlu*I for W52A; *Xma*I for M114A, Q115A/E, and Y116A). The resulting PCR fragments were digested with the appropriate restriction endonucleases and ligated into *MHelppUC19* cleaved with *Sac*I and *Eco*72I. Following sequence verification, the *Sac*I-*Eco*72I fragment of *MHelppUC19* was ligated into *pSARM4* (kindly provided by E. Hunter). For single-round infectivity assays, the *Sac*I-*Eco*72I fragment of *MHelppUC19* was ligated into the *pSARM-EGFP* vector, in which enhanced green fluorescent protein (EGFP) replaces the *env* gene [28] (kindly provided by E. Hunter). To prepare vectors for expression of M-PMV CA-NTD or CANC proteins with A4Hel5, R14, D111, and Q115 mutations, the *Nde*I-*Xho*I fragments encoding CA-NTD or CANC, respectively, obtained by PCR using pSARM4 with the appropriate mutation as a template, were ligated into pET22b cleaved with *Nde*I and *Xho*I. Further details of the cloning strategy and full sequences of all PCR primers can be obtained from the authors upon request.

### Cell growth and virus production

HEK 293 T cells were grown in Dulbecco’s modified Eagle medium (DMEM, PAA Laboratories, Linz, Austria) supplemented with 10% fetal bovine serum (PAA) and 1% L-glutamine (PAA). Typically, HEK 293 T cells were plated at a density of 3 × 10^5^ cells/ml one day before transfection and then transfected with WT or mutant proviral DNA using X-tremeGENE DNA transfection reagent (Roche Molecular Biochemicals) according to the manufacturer’s instructions. Supernatants were harvested 48 h post-transfection, filtered through a 0.45 μm filter, and centrifuged through a 20% sucrose cushion for 1 h at 200,000 × *g* in a Beckman SW41Ti rotor.

### Protein expression, radioactive labeling, and quantification of particle release

HEK 293 T cells transfected with appropriate DNA were grown for 48 h post-transfection, starved for 30 min in methionine- and cysteine-deficient DMEM (Sigma), and pulse-labeled for 30 min with 125 μCi/ml of Tran^35^Slabel (M.G.P., Czech Republic). The labeled cells were then chased in complete DMEM for 4 h. Cells from pulse and pulse-chase experiments were washed with phosphate-buffered saline (PBS), lysed in 1 ml of lysis buffer A (1% Triton X-100, 1% sodium deoxycholate, 0.05 M NaCl, 25 mM Tris, pH 8.0) on ice for 30 min and clarified by centrifugation at 13,000 × *g* for 1 min. The culture medium of the chased cells was filtered through a 0.45 μm filter, and SDS was added to a final concentration of 0.1%. Viral proteins were immunoprecipitated from the cells and culture media with a polyclonal rabbit anti-M-PMV CA antibody and separated by sodium dodecyl sulfate polyacrylamide gel electrophoresis (SDS-PAGE). Radiolabeled proteins were visualized on a Typhoon Phosphorimager. To measure particle release, the radiolabeled protein bands of pulse–labeled [^35^S]-Gag (Pr78) and pulse-chase-labeled virion-associated CA proteins were quantified using ImageQuant TL (Amersham Biosciences). The released viral proteins were pelleted through a 20% sucrose gradient at 40,000 rpm in a Beckman SW41Ti rotor for 1 h. The virus release is shown as the relative concentration of CA correlated to the level of intracellular Gag in individual samples.

### Western blotting

HEK 293 T cells were transfected with WT or mutant proviral constructs. Virions from culture supernatants were harvested 48 h post-transfection as described above. The cells were washed with PBS and resuspended in 2 × SDS protein loading buffer (PLB). The cultivation media were filtered through a 0.45 μm filter and centrifuged at 40,000 rpm in a Beckman SW41Ti rotor for 1 h. Cell- and particle-associated viral proteins were separated by SDS-PAGE, blotted onto a nitrocellulose membrane, and detected with polyclonal antibodies against M-PMV CA.

### Quantitative Western blotting

Virion quantification was carried out as previously described [23]. Briefly, the amount of M-PMV CA protein in individual viral samples was determined by comparing the CA protein band intensities with a standard curve prepared using purified M-PMV CA [29]. Samples of particles released into the cultivation media, obtained as described above, were separated by SDS-PAGE, transferred onto a nitrocellulose membrane, probed with appropriate antibodies, and developed using West Femto Chemiluminescent Substrate (Thermo Scientific) and a LAS-2000 imager. Protein band densities were quantified using ImageQuant TL (Amersham Biosciences).

### Single-round infectivity assay

Infectivities of WT M-PMV and the mutants were determined as described [23,30]. Briefly, HEK 293 T cells were co-transfected with WT or mutant *pSARM-EGFP* expression vector [28] and the glycoprotein expression vector *pTMO* [24]. At 48 h post-transfection, culture supernatants were collected and filtered through a 0.45 μm filter. Each sample was normalized for CA protein content by quantitative Western blotting. The volume of culture supernatant used to infect the HEK 293 T cells was adjusted such that equivalent amounts of virus were added to each sample, and the cells were incubated for an additional 48 h. The cells were then fixed with 4% formaldehyde, and the number of GFP-positive cells was determined using flow cytometry (BD FACSaria).

### Electron microscopy of bacterial and tissue culture cells

For thin-section electron microscopic (TEM) analysis of the assembly of M-PMV CANC in *E. coli* or WT or mutant M-PMV in HEK 293 T cells, visible pellets of cells expressing appropriate proteins were prefixed with freshly prepared 2.5% glutaraldehyde in 0.1 M cacodylate buffer, pH 7.5. After washing with 0.1 M cacodylate buffer, pH 7.5, the cells were postfixed in 1% osmium tetroxide, dehydrated in an ethanol series (30, 50, 70, 80, 90, and 100%), and embedded in fresh EMBED 812 or AGAR 100 epoxy resin. Ultrathin sections (70 nm) of cells were contrasted with uranyl acetate and lead citrate. A JEOL JEM-1200EX analytical transmission electron microscope operated at 60 kV was used for the analysis.

### Bacterial expression

Luria-Bertani medium containing 100 μg/ml ampicillin was inoculated with *E. coli* BL21 (DE3) cells carrying the appropriate construct. At an optical density at 590 nm of 0.8, expression was induced by the addition of isopropyl-B-D-thiogalactopyranoside (IPTG) to a final concentration of 0.4 mM. The cells were harvested 4 h post-induction.

### Protein purification for NMR spectroscopy

R14K and D111N CA-NTD^1–140^ were expressed and purified as previously described [6,31]. Briefly, the proteins were overexpressed in *E. coli* BL21 (DE3) and purified under native conditions by gel chromatography. To achieve ^13^C and ^15^N isotopic enrichment, the transformed bacterial cells were grown in M9 minimal medium supplemented with D-[*U*-^13^C]-glucose and [*U*-^15^N]-NH_4_Cl. The uniformly ^13^C, ^15^N-labeled CA-NTD^1–140^ sample for NMR spectroscopy was prepared in 50 mM Tris buffer, pH 8.0, containing 150 mM NaCl, 1 mM EDTA, 1 mM TCEP, and 5% deuterium oxide. The sample was concentrated to 1.0 mM in a volume of 500 μl.

### NMR spectroscopy

All NMR experiments were conducted at 298 K. A set of ^15^N-edited and ^13^C-edited NOESY spectra [32] of the D111N mutant of [^13^C; ^15^N]-CA-NTD^1–140^ was recorded on a Bruker Avance I 900 MHz spectrometer equipped with a 5 mm cryogenic ^1^H/^13^C/^15^N TXI probe head, and the HCCH-TOCSY spectrum [33] was recorded on a Bruker Avance I 700 MHz spectrometer equipped with a 5 mm cryogenic ^1^H/^13^C/^15^N TXI probe head. A set of standard 3D triple-resonance HNCA, HN(CO)CA, HNCACB, CBCA(CO)NH, and HNCO experiments [33], as well as all other experiments, were collected on a Bruker Avance III 600 MHz spectrometer equipped with a 5 mm cryogenic ^1^H/^13^C/^15^N TXI probe head.

All spectra were processed using the spectral processing and analysis program NMRPipe/NMR-Draw 3.0 [34] and analyzed using the graphical NMR assignment and integration software Sparky 3.111 (T. D. Goddard and D. G. Kneller, University of California, San Francisco, USA).

### Hydrogen-deuterium exchange

Hydrogen-deuterium exchange was monitored by NMR spectroscopy as a decrease in signal intensity in ^15^N-^1^H HSQC spectra. A sample of 1 mM [^13^C; ^15^N]-CA-NTD^1–140^ (500 μl) dissolved in H_2_O was freeze-dried. The freeze-dried sample was dissolved in [99.9% ^2^H]-H_2_O, and the first experiment was started within 15 min after dissolution. The measurement time of a single spectrum was 10 min. A series of spectra was recorded until the intensities ceased to change. To compare the exchange rates of WT and the R14K mutant, the intensity of the signals was normalized to the intensity of the signals of un-exchanged amide protons.

### In vitro cleavage

Cleavage of CA-NTD protein with M-PMV protease was performed as described previously [35]. Briefly, M-PMV R14K CA-NTD and WT CA-NTD purified proteins in storage buffer (50 mM Tris buffer, pH 8.0, 150 mM NaCl, 1 mM EDTA, 1 mM TCEP) were diluted with 50 mM phosphate buffer, pH 6.2, containing 300 mM NaCl and 0.01% 2-mercaptoethanol to a final concentration of 0.5 mg/ml. M-PMV protease (13 kDa form) was added to the reaction at the final concentration of 2 μM and incubation was carried out for 4 h at 37°C. The protein samples were then separated using SDS/PAGE gels and stained by Coomassie blue or transblotted onto a nitrocellulose membrane and developed immunochemically using polyclonal rabbit anti-M-PMV CA antibody.

## Additional file

Additional file 1: Figure S1. Superimposed ^1^H-^15^N HSQC spectra of WT (red), D111N (blue), and R14K (green) CA-NTD proteins. (A) Backbone resonances of the respective proteins are labeled. Side-chain resonances are not labeled and backbone resonances in the framed central part of the spectrum are labeled only in (B) for the reason of clarity. The residues of D111N and R14K mutants with largest combined chemical shift changes are underlined.
