# Supplementary material for: Stabilization of the β-hairpin in Mason-Pfizer monkey virus capsid protein– a critical step for infectivity
Source: Retrovirology. 2014 Oct 30;11:94. doi: 10.1186/s12977-014-0094-8 (PMC4219007; doi:10.1186/s12977-014-0094-8)
Supplement: Additional file 1: Figure S1. — Superimposed 1H-15N HSQC spectra of WT (red), D111N (blue), and R14K (green) CA-NTD proteins. (A) Backbone resonances of the respective proteins are labeled. Side-chain resonances are not labeled and backbone resonances in the framed central part of the spectrum are labeled only in (B) for the reason of clarity. The residues of D111N and R14K mutants with largest combined chemical shift changes are underlined. [file 12977_2014_94_MOESM1_ESM.pdf]

A

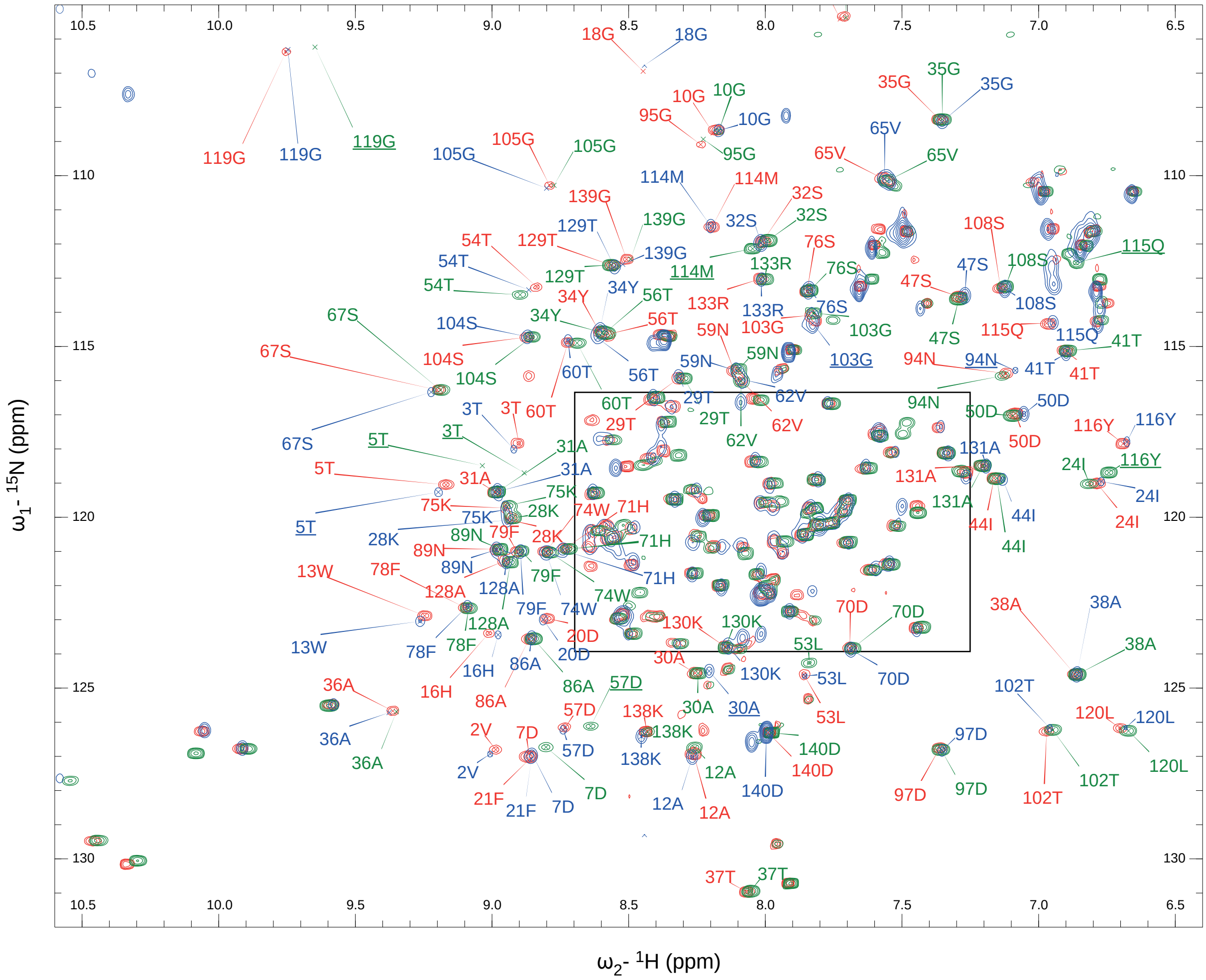
ω1- 15N (ppm)
ω2- 1H (ppm)
10.5
10.0
9.5
9.0
8.5
8.0
7.5
7.0
6.5
110
115
120
125
130

B

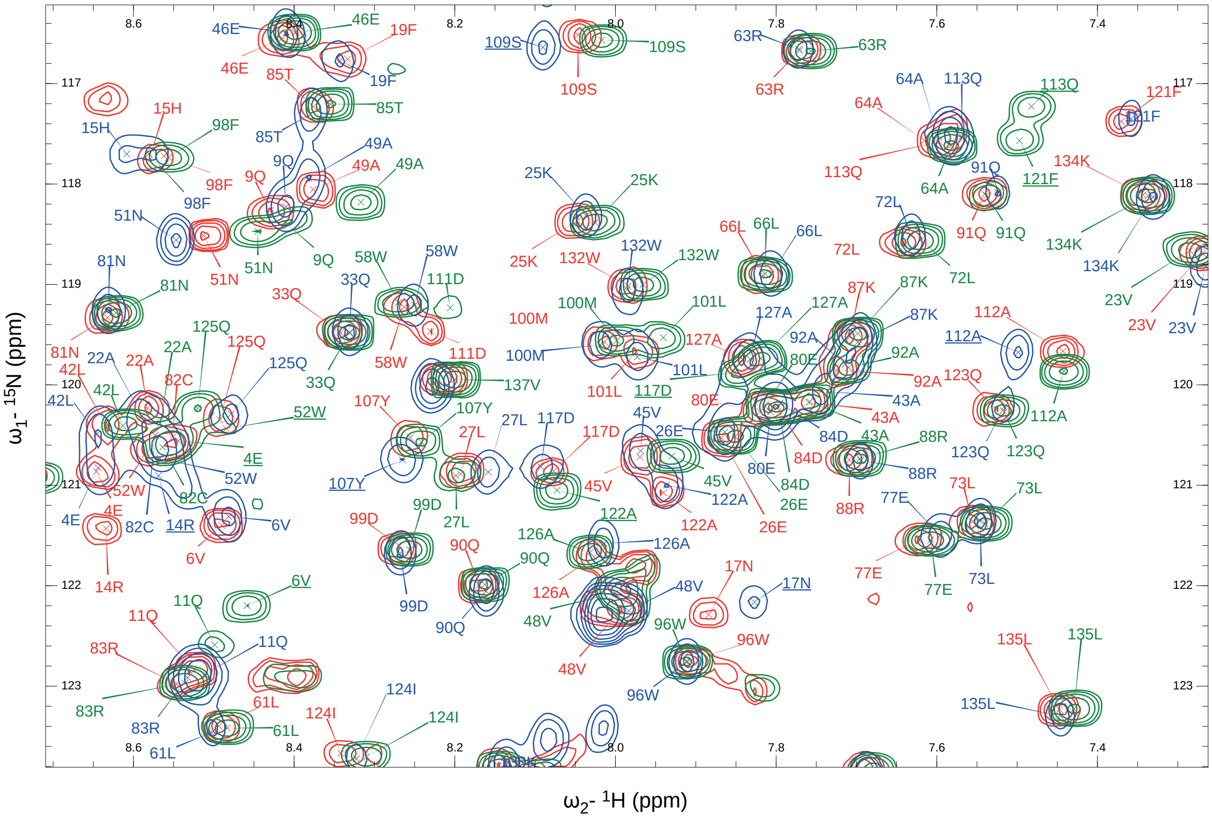
ω1- 15N (ppm)
ω2- 1H (ppm)
8.6
8.4
8.2
8.0
7.8
7.6
7.4
117
118
119
120
121
122
123
